# Partial immune responses in Sichuan bream (*Sinibrama taeniatus*) after starvation

**DOI:** 10.3389/fimmu.2023.1098741

**Published:** 2023-03-06

**Authors:** Jinfeng Shi, Dayou Zhuo, Minfang Lu, Haoyu Wang, Haoran Gu, Xiaohong Liu, Zhijian Wang

**Affiliations:** ^1^ Integrative Science Center of Germplasm Creation in Western China (Chongqing) Science City & Southwest University, Chongqing, China; ^2^ Key Laboratory of Freshwater Fish Reproduction and Development (Ministry of Education), Key Laboratory of Aquatic Science of Chongqing, Southwest University, Chongqing, China; ^3^ School of Life Sciences, Southwest University, Chongqing, China

**Keywords:** *Sinibrama taeniatus*, starvation, transcriptome, immune response, hematological parameters

## Abstract

**Background:**

Food deprivation is a severe stress across multiple fields and it might be a challenge to immune system.

**Methods:**

In the present study, adult male *Sinibrama taeniatus* were deprived of feed for 7 to 28 days. We explored the effects of starvation on immunity in *S. taeniatus* through hematological analysis, antioxidant capacity analysis, detection of the content or activity of immune factors in plasma, and transcriptomic analysis.

**Results:**

The results indicated that biometric indexes significantly decreased in the fish after starvation, the proportion of thrombocyte, neutrophil and monocyte increased and, conversely, the proportion of lymphocyte decreased. The antioxidant indexes (SOD and CAT) and innate immune parameters (LZM, C3) were upregulated in fish suffering from a short period of starvation, while adaptive immune parameter (IgM) conversely declined. The transcriptome analysis revealed the changes of various metabolic regulatory pathways involved in fatty acids and amino acids, as well as the immune responses and antioxidant capacity.

**Conclusions:**

Taken together, this research in the present study suggested an induced innate immunity while a partly suppressed adaptive immunity under a short period starvation.

## 1 Introduction

Basic life activities of all living organisms depend on the energy supply (1). Food fluctuation is a common and crucial stress in aquaculture due to the fluctuation during natural activities cycles such as fish reproduction, migration, *etc.* (2, 3). Especially for farmed fish, they may suffer from starvation due to underfeeding, uneven feeding or high breeding densities (4).

Most fish species cope with short-/long-term fasting when submitted to the environmental challenges by various physiological responses (5). Inevitably, changing of nutrition supply affects the function of the hematopoietic system and the immune system if the stress response is costly or the organismal compensation is imperfect (1, 3, 6). The innate immune response is the first boundary of host defense, protecting alive animals from pathogens, and adaptive immunity can mount a more targeted and advanced immune response (7). Fish immune organs are comprised of thymus, head kidney, trunk kidney, spleen, gills, liver and intestine, of which head kidney, trunk kidney, spleen and liver are also important hematopoietic organs (8). There are a variety of cell types involved in innate immune response, including monocytes (or macrophages), non-specific cytotoxic cells and granulocytes, and lymphocytes were mainly involved in specific immunity in fish. In recent reports, erythrocytes and thrombocytes of fish were reported to be functional in immune function as well (9, 10). Besides the immune cells, plasmatic antibodies, complements, antibacterial peptides and other immune factors constitutes the humoral immune system Among them, lysozyme, complement and tumor necrosis factor are mainly involved in innate immune response, while immunoglobulins produced by B cells are mainly involved in adaptive immunity. SOD and catalase are important components of antioxidant systems and they are also relevant to the fish immune capacity (11). Therefore, the analysis of blood can provide a wealth of information on the immune status of fish (12). Previous study has revealed the increase of red and white blood cells in Nile tilapia (*Oreochromis niloticus*) after a two-week starvation (13), as well as a maintained fast-responsive innate immune capacity in Atlantic salmon (*Salmo salar*) after a long period of fasting (14). However, what’s the situation in the adaptive immune system of aquatic fish after food deprivation, and the underlying mechanism are still unknown.

Sichuan bream (*Sinibrama taeniatus*) is a small economic fish endemic to the upper reaches of the Yangtze River. It provides important economic and ecological values in the local area (15). However, population of this fish shows continuous sharp decline due to excessive human activities, such as overfishing, abuse fishing, construction of cascades of dams across rivers. Artificial breeding and cultivation of Sichuan bream has been overcome recently in our lab, but biology of this fish still remains largely unknown. The recent preliminary work has shown that the hematopoietic organs of *S. taeniatus* are mainly comprised of head kidney, trunk kidney, spleen and liver. Among them, the trunk kidney contains a large number of both mature and immature immune cells in various stages. Thus, it is an important hematopoietic and immune organ of *S. taeniatus* (unpublish). In order to investigate the changes in both innate and adaptive immune systems caused by insufficient energy supply, and the further interaction between immune and metabolic mechanisms to reveal the underlying mechanism, *S. taeniatus* were challenged to fasting for 7 days (7d), 14d and 28d, and the biological indexes, hematological parameters and immune indexes of male *S. taeniatus* were detected in the present study, and the underlying mechanisms was also explored by transcriptome analysis.

## 2 Materials and methods

### 2.1 Fish and experimental conditions

210 healthy (without any symptoms such as hemorrhage, ragged fins, abdominal distension) yearling male *S. taeniatus*, with body weight of 12.36 ± 0.89 g and length 8.73 ± 0.49 cm were selected from the husbandry in Key Laboratory of Freshwater Fish Reproduction and Development, Ministry of Education, Chongqing City, China, under a water-circular system. The fish were domesticated in non-cyclic water for two weeks. During this time, they were fed to saturation on a commercial diet twice a day (at 9 a.m. and 5 p.m., respectively). After acclimating, the fish were divided into control and starved groups, two groups were divided into three subgroups (7 d, 14 d, and 28 d), with triplicate tanks per subgroup and 8 individuals in each tank. Fish in the control group were fed under the protocol used in the acclimation phrases, while the starved group was deprived of feed for 7 to 28 days. Fish in all subgroups were separately cultured under the same environmental conditions, and these subgroups were named C-7d, C-14d, C-28d, S-7d, S-14d and S-28d, respectively. Water quality was maintained daily by renewing 30% of the water (dissolved oxygen content greater than 7 mg/L, pH 7.5 ± 0.5), with a maintenance temperature of 26 ± 0.5°C. The photoperiod was set as a 12 h: 12 h light-dark cycle.

### 2.2 Determination of biometric indexes

During the starvation period, fish were sampled and measured at 7, 14, 28 days. In brief, after anesthetized with MS-222 (500 mg/L, Sigma Chemicals Inc., USA), the body mass and body length of each fish in each group were measured. Biometric indexes including weight gain percentage (WG, %), condition factor (CF, g/cm^3^), visceral somatic index (VSI, %) and hepatic somatic index (HSI, %). The above mentioned measurements were calculated as follows: WG% = (final body weight − initial body weight)/initial body weight×100, CF = Weight of fish (g)/(fish total length)^3^(cm)^3^ × 100, VSI = (total weigh of all viscera/total body weight) ×100 and HSI = (liver weight/total body weight) ×100 (16). The analysis was based on the number of replicate tanks (n=3).

### 2.3 Sample collection

Sample collection was performed on the 7d, 14d, and 28d after the starvation of the fish (17). At each sampling time, blood of 9 fish from each group were collected from the caudal vein. The small amount of blood (30 μL) was separately diluted by red and white blood cell dilutions (Solarbio, Beijing, China, R1010) for counting of red blood cells (RBCs) and white blood cells (WBCs), another small part (2 μL) was used to blood smear for differential leucocytes counts (DLC). The rest of blood was used for plasma isolation, performed as centrifugation at 4000 rpm for 10 min at 4°C. Isolated plasma from each fish was stored at -20°C until use.

After blood collection, trunk kidney of three randomly chosen fish from each group were collected, snap-frozen in liquid nitrogen and then stored at −80°C until it was used for transcriptome sequencing and quantitative real-time PCR qRT-PCR validation experiments.

### 2.4 Analysis of antioxidant activities(SOD and CAT)

The superoxide dismutase (SOD, A001-3-2) activity and the catalase (CAT, A007-1-1) activity of plasma samples were determined using commercial test kits (Nanjing Jiancheng Bioengineering Institute, Jiangsu, China). Both measures were expressed as units per milliliter (U/mL).

### 2.5 Detection of immune parameters (LZM, C3, TNF-α and IgM)

The plasma lysozyme (LZM) (U/L), complement 3 (C3) (ug/mL), tumor necrosis factor-α (TNF-α) (pg/mL) and immunoglobulin M (IgM) (ug/mL) were measured using ELISA kits (Jiangsu Meimian Industrial Co., Ltd, Jiangsu, China). The LOT numbers of these four kits were MM-925409O1, MM-33667O1, MM-0655O1and MM-33677O1 respectively. These immune parameters were determined according to the manufacturer’s instructions, in a microplate reader (Thermo Scientific Varioskan Flash, USA) at 450 nm to measure the optical density.

### 2.6 Kidney transcriptome analysis

Trizol Reagent was used to extract the total RNA from the kidney tissues of *S. taeniatus* in 6 subgroups following the manufacturer’s instructions. The purity of sample RNA was checked using the Nanodrop (OD 260/280), and the concentration was measured using Qubit^®^ RNA Assay Kit in Qubit^®^2.0 Flurometer (Life Technologies, CA, USA), and the integrity was assessed using the Agilent 2100.

The sequencing libraries were constructed with the TruSeq PE Cluster Kit v3-cBot-HS (Illumina). The library products were analyzed and sequenced on an Illumina Hiseq platform. After elimination of raw reads, paired-end clean reads were aligned to the reference genome of *S. taeniatus* (unpublished) using STAR (http://code.google.com/p/rna-star/). And then FPKM of each gene was calculated based on the length of the gene and reads count mapped to this gene. The differentially expressed genes (DEGs) between the starvation group and the control group were evaluated by FPKM with an adjusted P <0.05 and absolute Log2 fold change (|log2 FC| > 1). Gene Ontology (GO) and KEGG pathway enrichment analyses of differentially expressed genes were implemented by the cluster Profiler R package (http://bioconductor.org/packages/release/bioc/html/clusterProfiler.html). P<0.05 was used as the statistically significant enrichment of both GO and KEGG (18). The raw transcriptome data was available at BioProject PRJNA902429.

### 2.7 Weighted gene co-expression network analysis

WGCNA is a robust algorithm highlighted by the modular clustering of genes and the association analysis between the modules and clinical traits (19). In the present study, WGCNA was conducted to analyze all DEGs in different stages of the mRNA expression data using the R package “WGCNA” (http://www.genetics.ucla.edu/labs/horvath/CoexpressionNetwork/Rpackages/WGCNA). First, a sample-clustering tree was formed to assess the presence of outliers. Then, the adjacency matrix was constructed using a soft threshold power of 30. Network interconnectedness was measured by calculating the topological overlap using the TOMdist function with a signed topological overlap measure (TOM) type. After that, the genes that had similar expression levels were clustered in modules and were used in subsequent analysis. To reveal the correlation and underlying mechanisms, Pearson’s correlation analysis was used to calculate the relationships between gene modules and the above tested innate and/or adaptive immunity indexes.

### 2.8 Validation of RNA-seq profiles by qRT-PCR

To validate the RNA-seq results, 14 DEGs were selected from the transcriptome data for qRT-PCR analysis. The cDNAs were synthesized in a 20 μL reaction volume containing 1 µg total RNA, using a reverse transcription kit (Takara, China). The forward and reverse primers (**Supplemental Table 1**) were designed based on the genomic and transcriptomic sequences using Primer Premier 5 software (Premier Biosoft, California, USA). The qRT- PCR was performed with SYBR Green ExTaq II kit (Takara, China) and StepOne™ real-time PCR system (ABI, New York, USA). Beta-actin was used as the internal reference. The amplifications were conducted in a 10 μL reactions, which contained 5 μL master mixes, 1 μL of cDNA, and 0.25 μL of each primer. The qRT-PCR reactions were conducted with the following procedure: 95°C for 10min, then 40 cycles of 95°C for 15 s, 60°C for 60s. Gene expression levels were calculated with the 2^−ΔΔCt^ approach (20).

### 2.9 Statistics

Bioassays were replicated at least three times, and laboratory personnel participated in the study were blinded to treatment assignment. Statistical analyses were performed using SPSS 21.0 software (SPSS, Chicago, IL, USA), and Prism 6 (GraphPad Software, San Diego, CA) was used for graphics. Significant differences among starved and control groups were determined using two-tailed t-tests with significance at P< 0.05. Each variable value was expressed as the mean ± standard error (SEM).

## 3 Result

### 3.1 Biometric indexes

**Figure 1:**
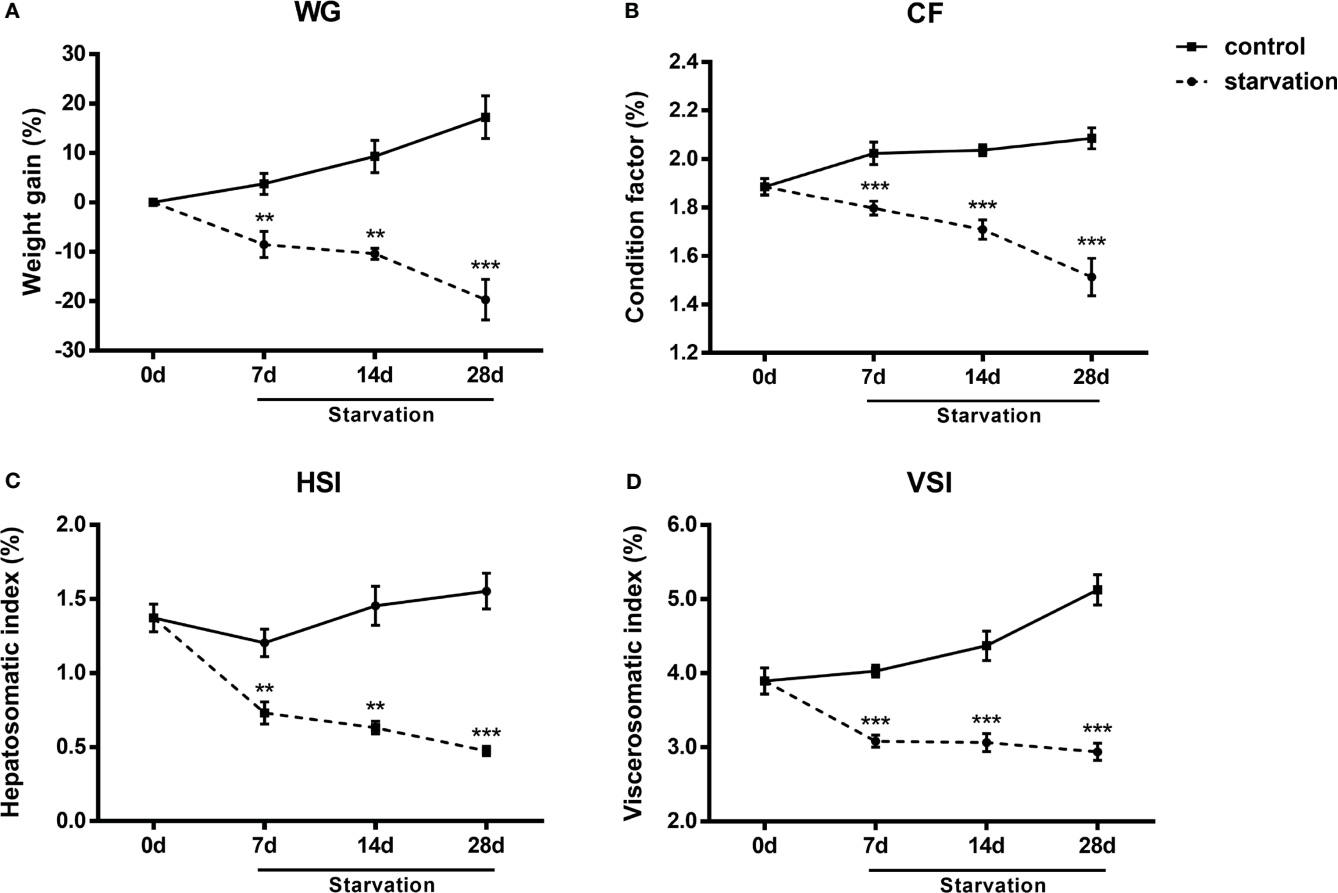
The changes of biometric indexes in *S. taeniatus* under starved conditions. **(A)** weight gain percentage (WG, n=3 replicate tanks); **(B)** condition factor (CF, n=24 individuals); **(C)** visceral somatic index (VSI, n=9 individuals); **(D)** hepatosomatic index (HSI, n=9 individuals). **P<0.01, ***P<0.001.

No mortality occurred during the 28 days of starvation treatment. WG, CF, VSI and HSI exhibited similar reduction tendency and all these indexes were significantly decreased after 7 days of starvation (P< 0.05, **Figures 1A–D**).

### 3.2 Hematological parameters

**Figure 2:**
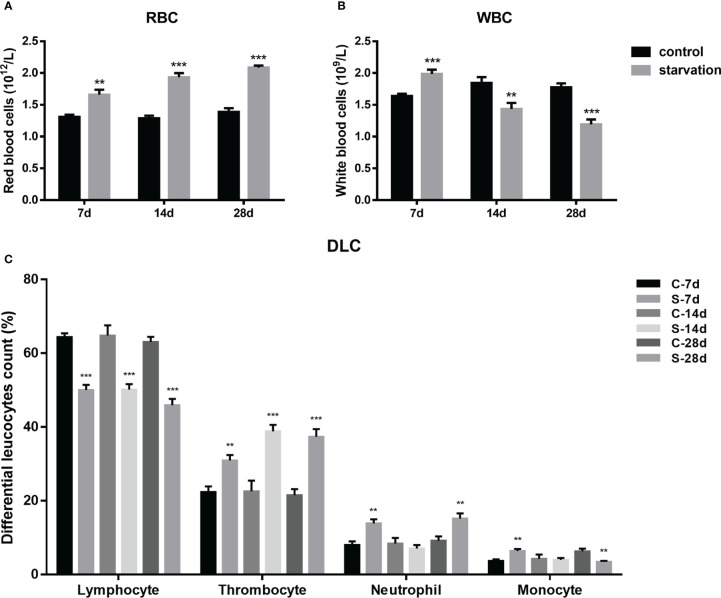
The impact of starvation stress on hematological parameters in *S. taeniatus*. **(A)** red blood cells (RBCs, n=9); **(B)** white blood cells (WBCs, n=9); **(C)** differential leucocytes count (DLC, n=12). (C-7d represents the control group after 7 d of feed, S-7d stand for starved group after 7d of starvation, other groups are named in the same way as this one). **P<0.01, ***P<0.001.

RBCs were significantly raised in all starved fish (P< 0.01, **Figure 2A**), with a time dependent manner. As shown in **Figure 2B**, WBCs of starved fish was obviously higher than the control group at 7 days, while declined at 14 and 28 days of starvation (P< 0.01).

According to our previous research, peripheral leukocytes of *S. taeniatus* were mainly comprised of four types including lymphocytes, thrombocytes, neutrophils, monocytes and monocytes with decreasing number in turn. The proportion of these four leukocyte types showed change significantly after starvation.

As shown in the **Figure 2C**, starvation for 7, 14 and 28 days caused significant decrease of the proportion of lymphocytes (P<0.001). It significantly decreased by 14.34% after 7 days of starvation compared to the control group (P< 0.05). When starved for 28 days, the proportion only accounts for 45.89 ± 5.77% (P< 0.05). However, the proportion of thrombocytes significantly increased in starved groups, by degrees from 30.91 ± 4.91% to 37.34 ± 6.94% (7 day- 28 days) (P< 0.01). The proportion of neutrophils increased by 5.82% and 5.99% after 7 and 28 days of starvation respectively, while recovered to control level at 14 days. The proportion of monocytes showed a similar alteration pattern with neutrophils within 14 days of starvation, but significantly decreased after 28 days of starvation (P< 0.01).

### 3.3 Antioxidant activity in the plasma

**Figure 3:**
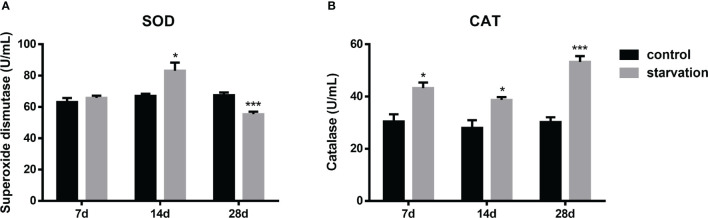
The effects of starvation on antioxidant activity in *S. taeniatus* (n=6). **(A)** superoxide dismutase (SOD); **(B)** catalase (CAT). *P<0.05, ***P<0.001.

The SOD activities increased significantly after 14 days of starvation but decreased after 28 days of starvation (P < 0.05, **Figure 3A**). CAT levels were consistently and significantly higher than those in the control group (P< 0.05, **Figure 3B**).

### 3.4 Determination of immune parameters in the plasma

**Figure 4:**
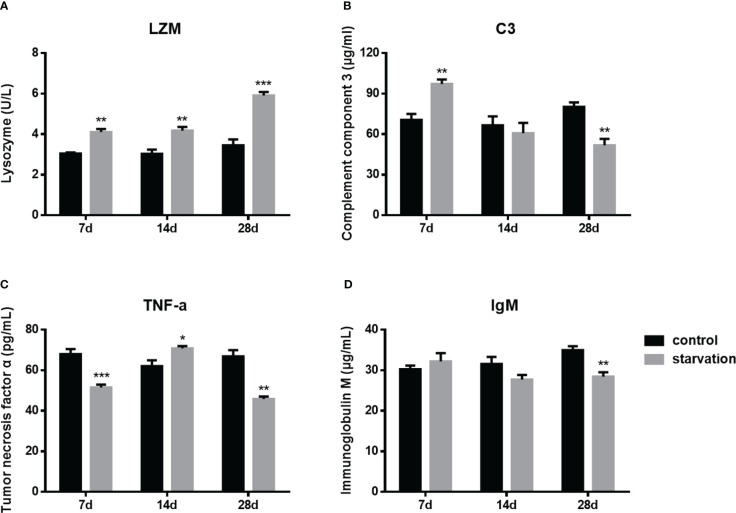
The changes of immune parameters in *S. taeniatus* (n=6). **(A)** lysozyme (LZM); **(B)** complement component 3 (C3); **(C)** tumor necrosis factor α (TNF-α); **(D)** immunoglobulin M (IgM). *P<0.05, **P<0.01 and ***P<0.001.

During the entire starvation period (from 7 day to 28 days), the plasma lysozyme activity presented a significant raise trend (P< 0.01, **Figure 4A**). After 7 days of starvation, the C3 levels was significantly higher than that of the control group, while it was significantly lower than control group for 28 days starved (**Figure 4B**). However, the trend of TNF-α was different from the above two indexes. It was higher than the control group only after 14 days of starvation, while significantly lower in both 7-day and 28-day starvation (**Figure 4C**).

As an immunoglobulin IgM plays an important role in the adaptive immunity of fish. No significant changes were detected in the early stage of starvation (7 day- 14days), while it decreased significantly after 28 days of starvation (P<0.01, **Figure 4D**).

### 3.5 Functional classification of differentially expressed genes

**Figure 5:**
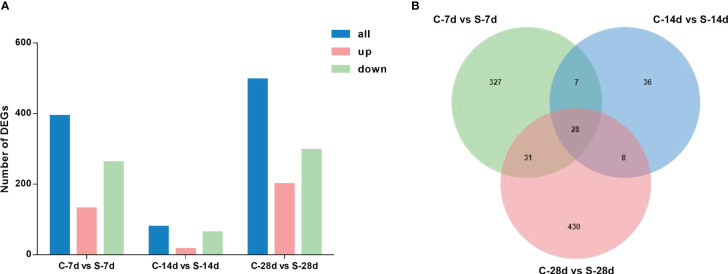
DEGs in the kidney of from *S. taeniatus* under starvation stress. **(A)** The number of DEGs; **(B)** The Venn diagram of unique and shared DEGs of *S. taeniatus* under starvation stress.

The 7-day starvation resulted in 393 DEGs in the kidney of *S. taeniatus*, with 131 up-regulated genes and 262 down-regulated ones. The 14-day starvation resulted in 79 DEGs with 16 up-regulated genes and 63 down-regulated genes. The starved 28 days group resulted in 497 DEGs with 200 up-regulated genes and 297 down-regulated genes. In general, most of these genes were related to metabolic function, and they were mainly affected by starvation stress, such as *Hadha*, *ANGPTL4* and *Mgll* (**Figure 5A**). Interestingly, we noted some genes associated with immune response, such as *C3*, *IL1R1*, *ccl19*, *Gpx4*, *eomes* and *etc.* 28 DEGs were shared in these three different starvation periods (**Figure 5B**).

**Figure 6:**
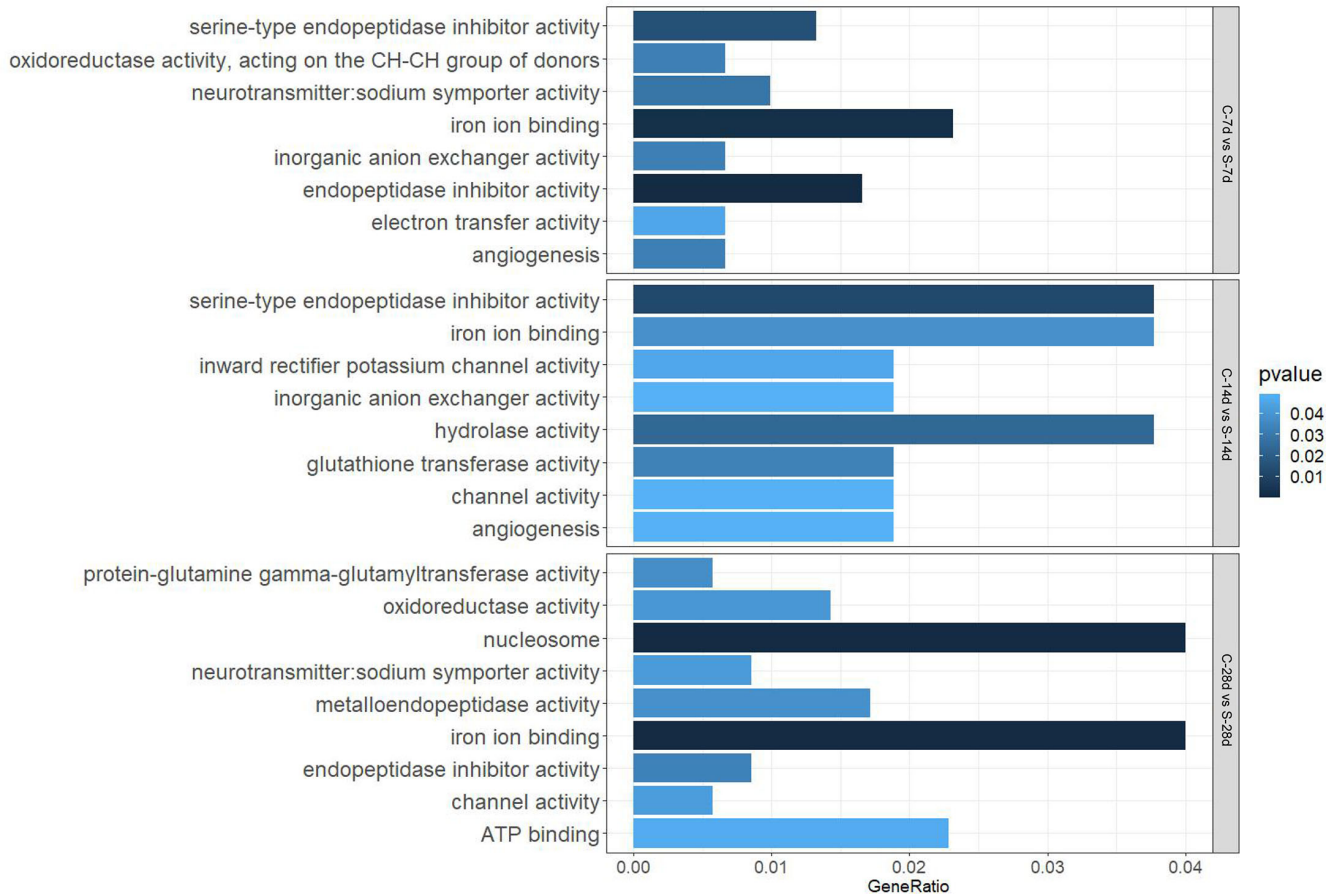
Enriched GO terms in the kidney of *S. taeniatus* after starvation for three time nodes.

GO analysis showed that starvation for both 7 days and 14 days mainly affected “biological processes” and “molecular function”, such as “iron ion binding”, “inorganic anion exchanger activity”, and there were 29 DEGs and 13 DEGs in the affected pathways, respectively. Starvation for 28 days mainly affected “cellular composition” and “molecular function” (60 DEGs), which involved biological processes such as “protein-glutamine gamma-glutamyltransferase activity” (no.1) and “oxidoreductase activity” (no.3) related to apoptosis as well as the antioxidant system (**Figure 6**).

**Figure 7:**
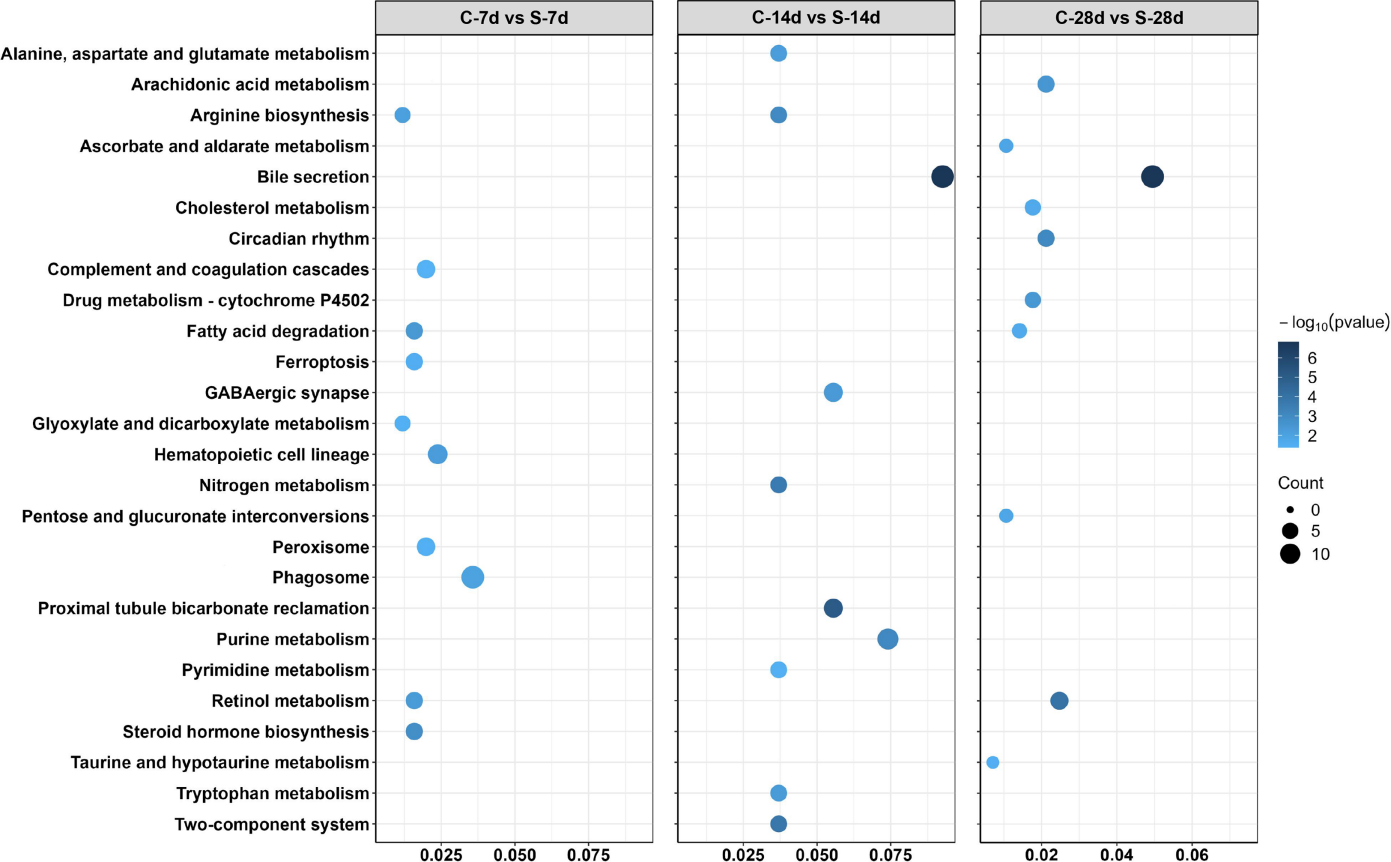
Enriched KEGG pathways in the kidney of *S. taeniatus* after starvation for three time nodes.

In addition, 51 different DEGs pathways of significant enrichment were identified with KEGG pathway analysis (**Figure 7**). Top 10 KEGG pathways at each of the phase were related to the digestion metabolism and mainly involved the energy-substance metabolism, including lipids, proteins, and amino acids. In addition, these pathways also involved signal transduction regulation, which was mainly related to immune response regulation such as “phagosome”, “complement and coagulation cascades” and so on.

### 3.6 WGCNA network construction and immune-related module identification

**Figure 8:**
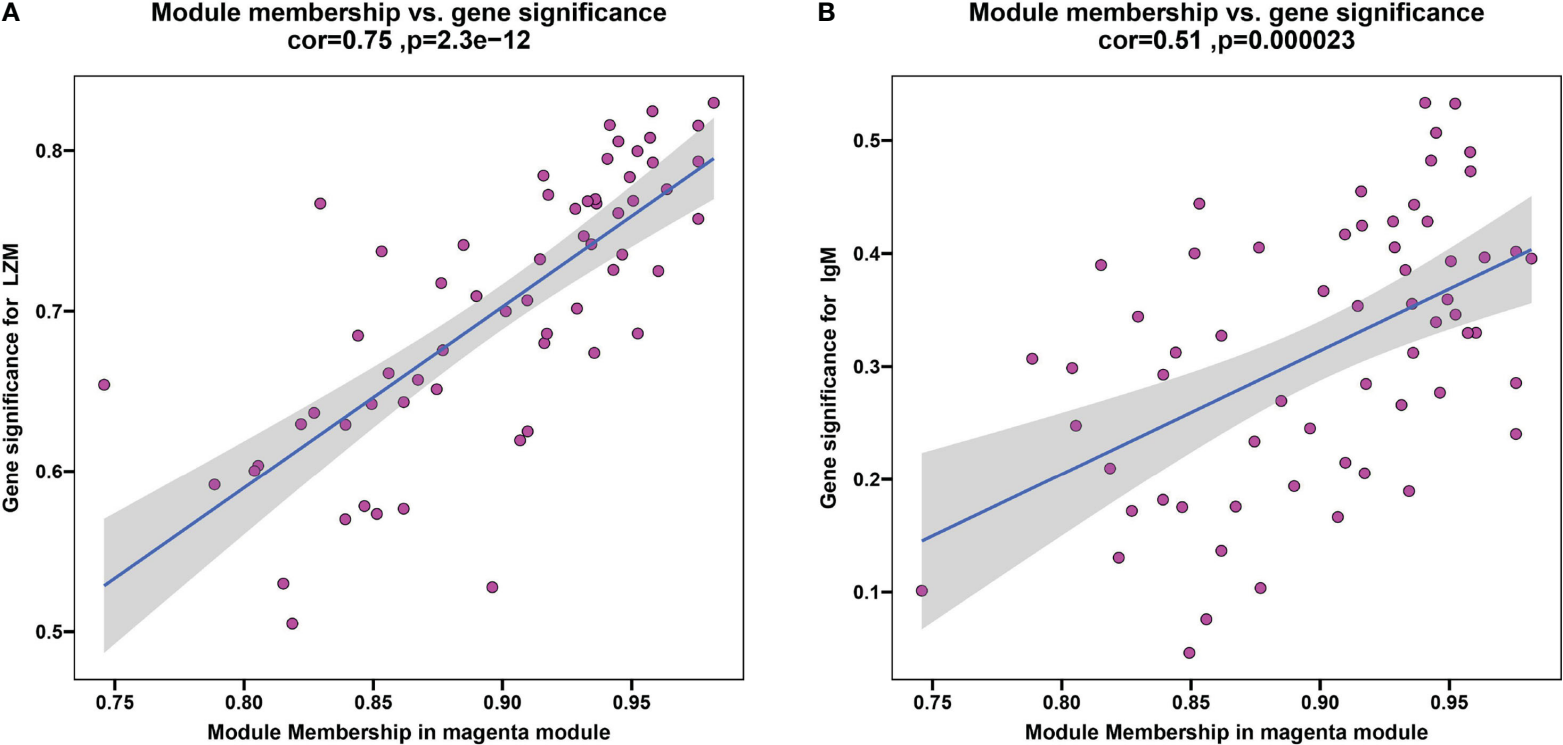
The result of WGCNA. **(A)** Plasma LZM activity correlated with module membership in magenta module; **(B)** Plasma content of IgM correlated with module membership in magenta module.

To discern if potential gene modules correlate with innate and adaptive immunity, plasma lysozyme activity and content of IgM, representing typical fish innate and adaptive immune indexes respectively, were selected as analysis traits here. A scale-free network was established with the WGCNA method, while a soft threshold was set to 30 by calculation (R^2^ = 0.90) (**Supplementary Figure S1**). A total of 9 modules were identified based on the traits of plasma LZM activity and content of IgM *via* average linkage clustering (**Supplementary Figure S2**), and the magenta module was the strongest correlated one. In particular, correlation values of the magenta module to LZM and IgM were 0.75 (P = 2.3e-12) (**Figure 8A**) and 0.51 (P = 2.3e-5) (**Figure 8B**), respectively. In the magenta module, genes including *Gfi1b* was downregulated in the trunk kidney of *S.taeniatus* after starvation, and genes such as transferrin receptor (*TFRC*), GATA binding protein (*gata1*) were also identified in this module (**Supplementary Table 3**).

### 3.7 Validation of RNA-seq data by qRT-PCR

**Figure 9:**
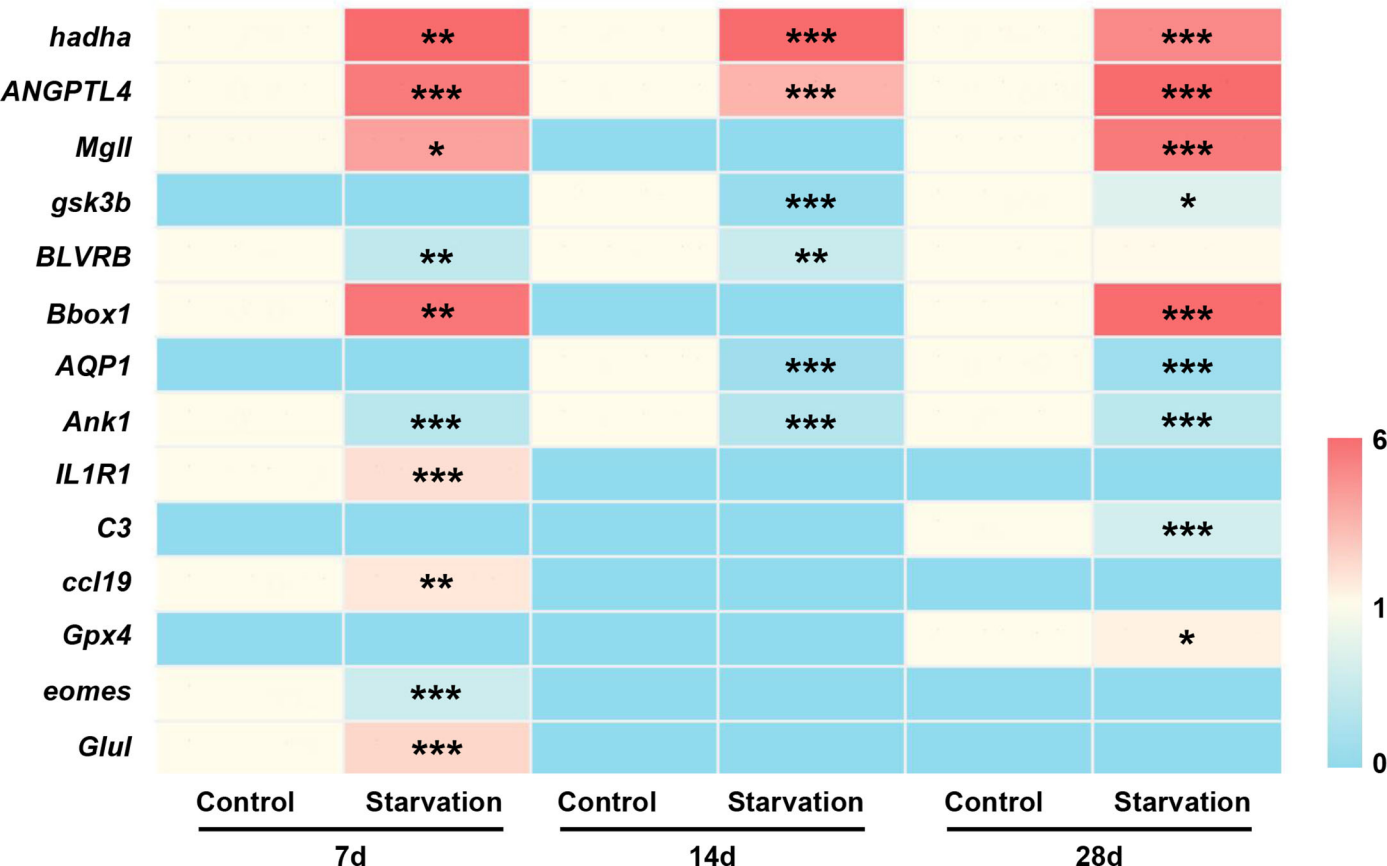
The gene expression of 14 selected DEGs of *S. taeniatus* under starved conditions, quantified by qRT-PCR. *P<0.05, **P<0.01 and ***P<0.001.

To verify the transcriptome sequencing data, 14 differentially expressed genes were selected for qRT-PCR confirmation. Among them, 8 genes (*hadha*, *ANGPTL4*, *Mgll*, *gsk3b*, *BLVRB*, *Bbox1*, *AQP1*, and *Ank1*) were related to metabolic function, and 6 genes associated with immune response (*IL1R1*, *C3*, *ccl19*, *Gpx4*, *eomes*, and *Glul*). qRT-PCR analysis showed that different length of starvation had different effects on gene expression, which was fully agreed with the results of RNA-seq (**Figure 9**).

## 4 Discussion

### 4.1 Starvation affected biometric indexes of *S. taeniatus*

When subjected to starvation, organisms developed integrated repertoire of physiological and biochemical responses that reduce metabolic expenditure and enhance endogenous substrates utilization, accompanied by loss of body mass and atrophy of tissues as well as downregulated function (21). The VSI and HSI are tightly related to the nutritional status and fat reserve of fish (22). In our present study, the sharp decline of these detected biometric parameters indicated the nutritional deficiency after starvation. Visceral organs are important energy deposition sites, they may function as key sources for energy supply during diet deprivation periods. The results were in consistent with previous studies on starved Nile tilapia (13), zebrafish (23) and rose snapper (*Lutjanus guttatus*) (24). Besides, our transcriptome data also showed that genes such as *Hadha*, *ANGPTL4* and *Mgll*, which were related to fatty acid metabolism were significantly up-regulated, while genes (including *Gsk3b*) related to glycogen synthesis were significantly down-regulated. This is in accordance with the viewpoint that lipids in visceral organs were fully used for energy supply and gluconeogenesis under starvation conditions to maintain basic physiological activities (25).

### 4.2 Starved effects on hematological indicators of *S. taeniatus*

Hematological indicators are sensitive to external environmental pressures (26, 27). In general, the main function of RBCs in fish is to carry and transport oxygen, and WBCs are mainly involved in cell defense and immune responses. In this study, RBCs in peripheral blood of fish increased with the extension of starvation time, while WBCs increased firstly and then decreased. In previous studies, RBCs and WBCs of Siberian sturgeon (*Acipenser baerii*) did not change significantly after 45 days of starvation (28). RBCs of grey mullet (*Mugil cephalus*) did not change significantly after starvation for 10-30 days, while WBCs increased after 10 days of starvation (16). However, RBCs and WBCs in Nile tilapia continuously raised within 7-21 days of starvation (13). Due to the different starvation tolerance of different fish species, there were certain differences in their hematological indicators after starvation. Raise of WBC has been supposed to be helpful in dealing with external pressure (29). For *S. taeniatus*, the first raise of WBCs at 7 days of starvation indicated a quick response capacity of the immune system in a short-term starvation state as well as the altered energy allocation in immune system.

From the perspective of DLC, the lymphocyte groups involved in specific immunity decreased significantly after starvation, accompanied with decreased IgM content, which is in accordance with the previous study that nutrient deprivation inhibited proliferation of peripheral blood lymphocytes (PBLs), resulting in reduced antibody production and specific immunosuppression (30, 31). The other two leukocyte groups, named neutrophils and monocytes, which were mainly involved in non-specific immunity were less affected. The proportion of monocytes decreased at 28 days, while increased at the early stage of starvation (7 days), and the proportion of neutrophils increased under all starvation periods. Thrombocytes, mainly involved in functions such as blood clotting but also in fish immune responses (10) were also significantly increased after starvation. The results of the present study are similar to the reported phenomena in sucker head (*Garra gotyla gotyla*) (26).

### 4.3 Starvation enhanced antioxidant activity

It is well known that SOD and CAT are important antioxidants in fish antioxidant defense system (32). SOD is a reliable indicator of oxidative stress, and its increased activity may be related to an increase of H_2_O_2_ production. CAT is a key antioxidant enzyme for H_2_O_2_ removal and a basic mechanism for limiting the formation of highly active hydroxyl radicals. CAT activity is mainly present in peroxisomes and is associated with elevated H_2_O_2_ concentrations as well (33). The results showed that SOD activity was the highest after the 14-day starvation (**Figure 3A**), indicating the increased SOD level of fish. However, when starved up to 28 day, the decreased SOD activity might be caused by excessive free radicals, and thus the damaged antioxidant system in the fish. In addition, the higher CAT activity also indicated that the fish was subjected to greater oxidative stress under starvation treatment within 28 days. Apart from this, in the KEGG pathways for DEGs, the redox system-related pathway (peroxisome) was enriched. This result showed that starvation caused oxidative stress in the fish body in a chronic manner. The same studies were seen in rainbow trout (*Oncorhynchus mykiss*) (34), European sea bass (*Dicentrarchus labrax*) (35), Soleimani (*Mesopotamichthys sharpeyi*) (36), brown trout (*Salmo trutta*) (37). Persisting oxidative stress can activate a large range of transcription factors, such as NF-κB, Nrf2 and HIF-1α. Subsequently, these transcription factors induce the expression of many cytokines and chemokines (38). In the trunk kidney tissues of the study, there are a large number of antioxidant-related genes such as *Gpx4* and innate immune-related genes such as *C3* and *ccl19*, which were all expressed up-regulated. Thus, oxidative stress can lead to chronic inflammation (38).

### 4.4 Starvation altered the immune response in *S. taeniatus*

LZM, C3, TNF-α are all important immune factors involved in innate immunity, and their activities or contents in plasma are good indicators of the innate immune response of fish (39). LZM can disrupt bacterial cell walls by catalyzing the hydrolysis of 1,4-β-bonds (40). C3 is the most important and core component in the complement system. It is worth mentioning that it is considered to be the main acute phase protein in response to external stimuli in vertebrate animals (41). TNF-α is an important activator of macrophages, by increase the respiratory activity, phagocytosis, and nitric oxide production of macrophages (41). Here we found a time dependent increase of LZM (**Figure 4A**), which was similar to the results of Chinese sturgeon (*Acipenser sinensis*) (42), Nile tilapia (13), grey mullet (16). C3 increased significantly in a short period of starvation (7d), and decreased significantly after 28 days of starvation (**Figure 4B**). Similarly, after 28 days of starvation, both transcriptome data and qRT-PCR results showed that the C3 gene was significantly down-regulated. TNF-α in plasma was significantly higher than that in the control group only after 14 days of starvation, while TNF-α gene expression detected in the spleen of hybrid grouper (*Epinephelus fuscoguttatus*♀×*E.lanceolatus*♂) (43) and the testis of zebrafish increased gradually with the extension of starvation (44). Therefore, as fish cope with environmental challenges, these innate immune factors are effective in stimulating inflammation (45). But the intricate influence of starvation stress and immune responses could be varied by the duration time and species or tissues (44). Combined with the increased proportion of thrombocyte, neutrophil and monocyte in the early stage of starvation, it can be inferred that short term starvation can promote the innate capacity of fish to a certain extent.

IgM is the most important medium in the specific immune response of fish (46). In the present study, plasma IgM content of *S. taeniatus* was unchanged within a starvation of 14 days, but significantly decreased at a longer time starvation (**Figure 4D**). Besides, based on the transcriptome analysis, genes associated with immunoglobulins were also significantly down-regulated after starvation (**Supplementary Table 2**). Decrease of serum IgM levels have been reported in fantail goldfish (*Carassius auratus* L.) after 8 weeks of fasting (47), and the similar downward trends were observed in channel catfish (*Ictalurus punctatus*) (48) and bastard halibut (*Paralichthys olivaceus*) (49) as well. However, there was still limited knowledge about the influence of insufficient energy supply upon adaptive immune system. The study has shown that mouse after dietary restriction were inhibited immune capacity due to reduce immunoreactivity to anti-immunoglobulin G antibody (50). These results suggested that starvation stress causes some degree of damage to the body’s adaptive immunity. In current study, combined with the decreased proportion of lymphocyte, the results here suggested an inhibitory effect of starvation on the specific immune response of fish.

### 4.5 Kidney transcriptome in response to starvation

In this study, the KEGG pathways for DEGs showed that starvation stress mainly affected the lipid metabolism of *S. taeniatus*, including fatty acid degradation, arachidonic acid metabolism, cholesterol metabolism and other pathways. This was in consistent with the findings in gibel carp (*Carassius auratus gibelio* var. CAS III) (51), hoplosternum littorale (*Teleostei, callichthyidae*) (52). Genes such as *Hadha* and *Mgll* were up-regulated related to fatty acid hydrolysis, while other genes related to fatty acid synthesis such as *ANGPTL4* and *Ank1* were down-regulated. Thus, starvation could induce mobilization of lipid reserves by accelerating lipid catabolism and inhibiting lipogenesis, and then promote the energy generation (53). Meanwhile, in the results of KEGG pathways, we noticed that after starvation for 14 days, a large number of amino acid metabolic pathways, such as alanine, aspartate and glutamate metabolism, arginine biosynthesis, tryptophan metabolism etc., were enriched. Lipids are reportedly a major source of energy and largely accumulate in the body and are of high caloric value, and proteins are mobilized only when both lipid and glycogen supplies are nearly depleted (54). Thus, both processes related to lipid and protein metabolism were disrupted in kidney of *S. taeniatus* treated by long-term starvation in the present study.

The antioxidant system played an important role in maintaining normal physiological activity and detoxification. In the present study, “peroxisome” pathway was enriched and genes associated with antioxidants included *Gpx4*, *GLUL*, *GSTK1* and *ect.* were up-regulated. In mammals that were on a restricted calorie intake a number of different isoforms of glutathione peroxidase were increased (55). *GSTK1* encoded glutathione-s-transferases that function in cellular detoxification (56). In this study, *GSTK1* expressed upregulation at 7 days of starvation. Interestingly glutathione-s-transferase activity was up regulated in rainbow trout following 3 weeks starvation (57). These results reflected that starvation could activate the antioxidant system.

The pathway “complement system and coagulation cascades” which has major roles in innate immune defense has components significantly enriched in starved fish. These genes related to innate immunity, such as C3, *IL1R1*, *ccl19*, *Ali3* and etc., were expressed differently. *C3* played a central role in the activation of the complement system. *IL1R1* was an important mediator involved in many cytokine-induced immune and inflammatory responses. *ccl19* played a role in normal lymphocyte recirculation and homing. *Ali3* affected the activity of proteolytic enzymes to protected to maintain homeostasis and benefit for the immune. In the study, *IL1R1*, *ccl19* and *Ali3* were significantly upregulated while *C3* was significantly downregulated after suffering 28d starvation stress. The past study suggested that the control of the complement cascade is complicated with different components being independently regulated when energy was insufficient (14). In the study, *C3* was downregulated, possibly due to long-term lack of energy supply to the starved fish (2), but other innate immune-related cytokines were activated after starvation stress (44).

In current study, we identified some key genes that suppress adaptive immunity when subjected to chronic starvation stress. For example, antigen presentation-related *HLA-DPA1* expression was up-regulated, gene *Eomes*, cell marker from thymic precursors of self-specific memory-phenotype CD8 T cells+, CD276, which was involved in regulating the T cell-mediated immune response (58), and related genes as part of the immunoglobulin complex such as *IGHV3-21*, all of which were down-regulated after starvation. (**Supplementary Table 2**). The past study on humans have found that starvation can affect stimulation of B cells and secretion of cytokines of PBLs and cause a partial immunodeficiency (30). In addition, the study has reported Balb/c mouse after suffering starvation failed in the recovery of the functional abilities of T-helper cells (Th) and/or in expanding pools of memory B cells (59). In the WGCNA analysis, the gene *Gfi1b* was identified (**Supplementary Table 3**). *Gfi1b* is expressed during hematopoiesis and lymphopoiesis and is a key gene for early regulation of B lymphocytes and T lymphocytes (60). After starvation stress, *Gfi1b* was significantly downregulated in the trunk kidney of *S. taeniatus*, which may explain the decrease in the proportion of lymphocytes in DLC analysis (61). Malnutrition inhibited acquired immunity leading to depletion in lymphocytes and alteration of their functions (50). Combined with downregulation of genes associated with the immunoglobulin domain in the modules, the suppressed acquired immunity of *S. taeniatus* after starvation was further verified.

In general, the expression levels of these DEGs associated with the innate immune system were up-regulated in the early stage of starvation stress (7d), but their expression levels decreased after starvation for too long (28d). Mounting an immune response requires energy and an increase in metabolic activity, and the effectiveness of the response may be related to body energy reserves (62). During the early stage of starvation, the stored lipid can provide sufficient energy to mount an improved innate immune response for a rapid defense to the external pathogens invading. However, with the prolongation of starvation, the energy supply in the fish is insufficient and thus the immune level decreases. This is in agreement with previous studies that starved fish attempt to increase expression of several key immune related genes to maintain good health (2, 14). However, DEGs associated with adaptive immune responses begin to be downregulated after starvation, presented as the significant decrease of lymphocyte proportion.

## 5 Conclusion

In summary, our study explored the starvation stress on immune respond in Sichuan bream. Based on the biometric and transcriptome data, it is obvious that energy metabolism of Sichuan bream was disrupted by starvation. However, accompanied with the robust decrease of biometric parameters and disrupted lipid and protein metabolism, capacity of innate immunity was also induced, especially at the early stage of starvation, presented as the increased proportion of thrombocyte, neutrophil and monocyte and raised plasmatic content of LZM and C3. On the contrary, the decreased proportion of lymphocyte, plasma IgM and declined expression of related genes suggested an adverse inhibitory pattern of adaptive immunity. The present study will help us to reveal the survival strategy under stress, and thus provides us a hint in understanding the energy allocation between metabolism and immunity. However, more researches are needed for uncovering of underlying mechanisms.

## Data availability statement

The data presented in the study are deposited in the NCBI BioProject repository, accession number PRJNA902429.

## Ethics statement

The animal study was reviewed and approved by the Committee of Laboratory Animal Experimentation at Southwest University, Chongqing, China. Written informed consent was obtained from the owners for the participation of their animals in this study.

## Author contributions

JS, ZW, and XL conceived the ideas and designed this study. JS wrote the original manuscript and completed data analysis. JS, DZ, ML, and HW completed experiments. HG directed experimental operation. ZW and XL participated in the discussion of the experimental results and revision of the manuscript. All authors contributed to the article and approved the submitted version.
