# Supplementary figures and images for: Partial immune responses in Sichuan bream (Sinibrama taeniatus) after starvation
Source: Front Immunol. 2023 Mar 6;14:1098741. doi: 10.3389/fimmu.2023.1098741 (PMC10025346; doi:10.3389/fimmu.2023.1098741)

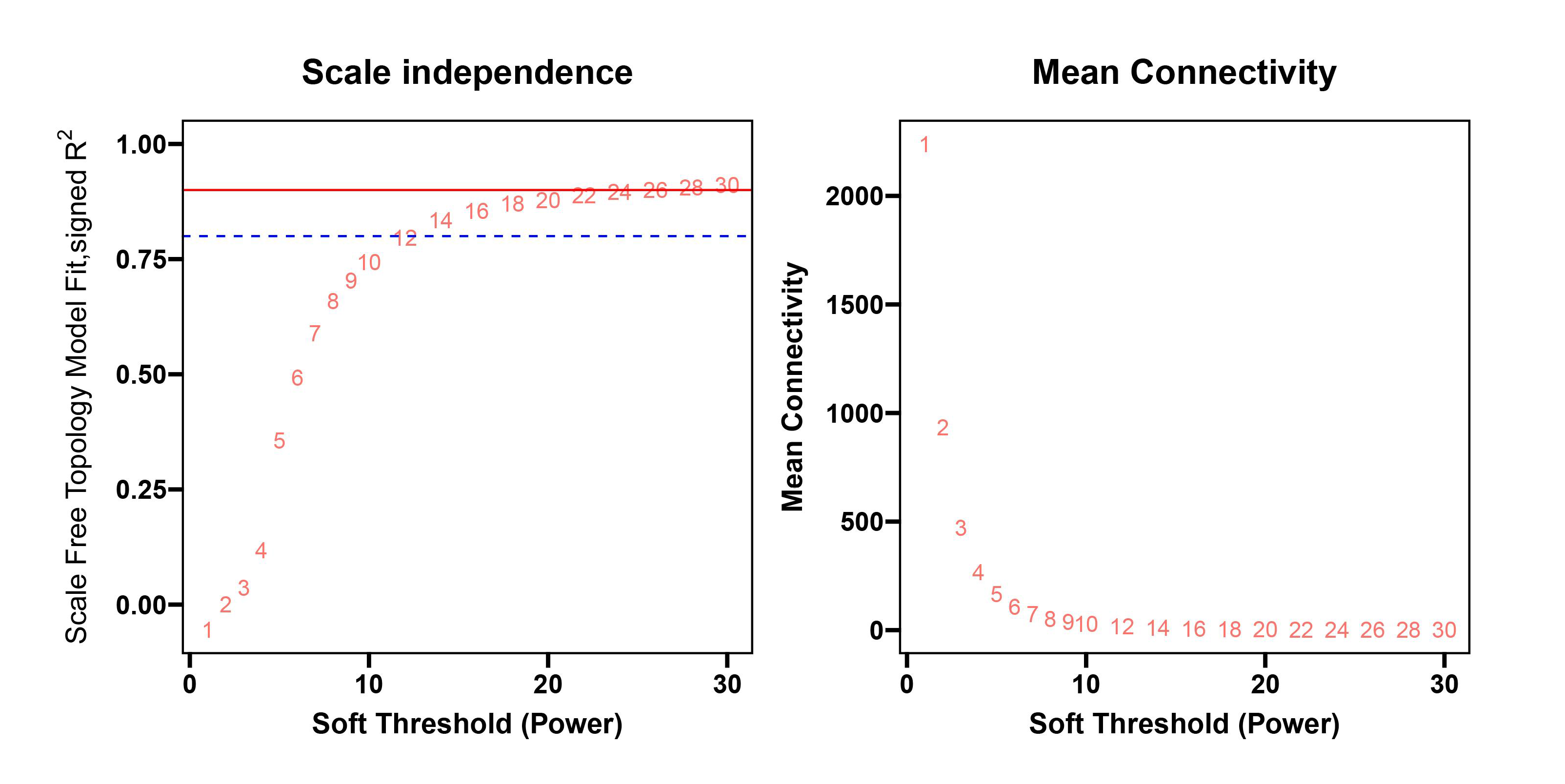

Supplement: Supplementary file 1 [file Image_1.tif]

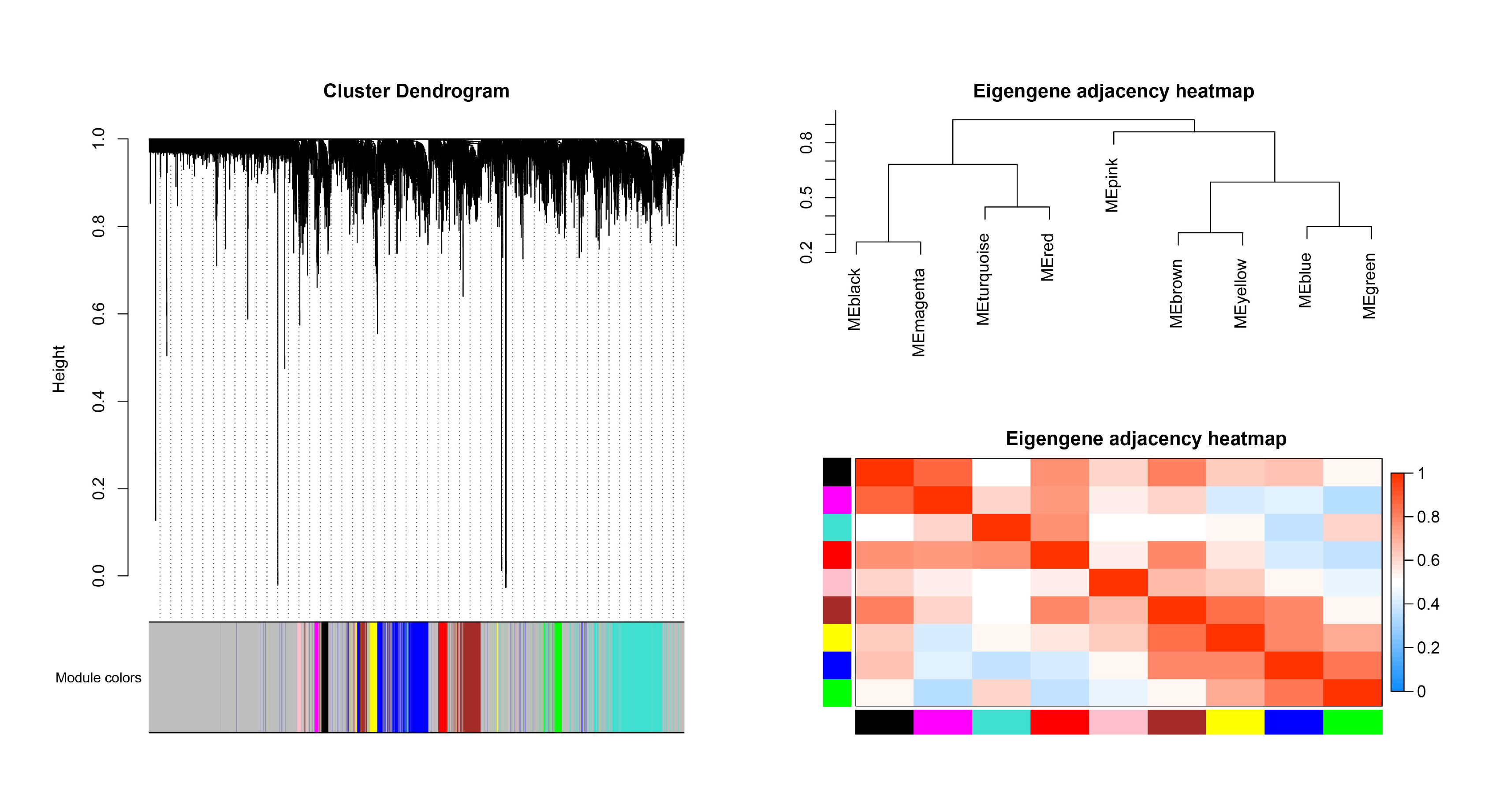

Supplement: Supplementary file 2 [file Image_2.tif]
