# Supplementary material for: Partial immune responses in Sichuan bream (Sinibrama taeniatus) after starvation
Source: Front Immunol. 2023 Mar 6;14:1098741. doi: 10.3389/fimmu.2023.1098741 (PMC10025346; doi:10.3389/fimmu.2023.1098741)
Supplement: Supplementary file 3 [file Table_1.docx]

**Supplementary** Tab.1

|  | **Gene name** | **Sequence (5'-3')** | **Length(bp)** | **amplification efficiency (%)** |
| --- | --- | --- | --- | --- |
|  | *β-actin*-F | CGAGCTGTCTTCCCATCCA | 86 | 94.01 |
|  | *β-actin*-R | TCACCAACGTAGCTGTCTTTCTG |  |  |
|  | *C3*-F | CATGGAGCGCCGAAATGAAG | 124 | 101.75 |
|  | *C3*-R | TGTCAGCCATGTGCTACTCG |  |  |
|  | *Gpx4*-F | ATCCGTGACTGTGAAGTC | 114 | 98.46 |
|  | *Gpx4*-R | CATCGCGTCTTGCTGTTCG |  |  |
|  | *IL1R1*-F | TTTGAGTTGGACCCTGGAGC | 171 | 104.50 |
|  | *IL1R1*-R | TGTAGTCTGGGCAGCGAAGTAG |  |  |
|  | *Ccl19*-F | AGCATTGGACTGTTGCCTGAC | 164 | 91.59 |
|  | *Ccl19*-R | ACTGCTCTTTAGTGACAGGTGGG |  |  |
|  | *GLUL*-F | CGTTCTCAAATACAACCGCAAA | 234 | 100.72 |
|  | *GLUL*-R | GCTTCTACAACATCTCGCCCG |  |  |
|  | *eomes*-F | ACTGGGCTACTACCCCGACTC | 294 | 95.56 |
|  | *eomes*-R | GGTGAAACACGCCTCCTCTTAC |  |  |
|  | *GSK3B*-F | GACAACCAATTTTCCCTGGTGA | 251 | 97.76 |
|  | *GSK3B*-R | TAGTGGGGTCAGTCGAGCTGTT |  |  |
|  | *hadha*-F | TGCCCAGGTAACAGTAGACAAGG | 296 | 96.16 |
|  | *hadha*-R | TGGTGGCAAAGATACAGTGAGG |  |  |
|  | *ANGPTL4*-F | CTGTGAAATGACACCCGAAGG | 120 | 94.16 |
|  | *ANGPTL4*-R | AACTCACCATCCAGGCTCCC |  |  |
|  | *Ank1*-F | CGGTAGCAAGTCCCATCCAC | 186 | 104.96 |
|  | *Ank1*-R | CCAAAGGGGGCGGTGTAG |  |  |
|  | *AQP1*-F | GGTTGTCCATTGCCACTCTTG | 187 | 98.50 |
|  | *AQP1*-R | TCTCCTTTCGTGACGCCATAC |  |  |
|  | *Mgll*-F | CTTGTTCTGCCGTTACTGGGA | 116 | 96.36 |
|  | *Mgll*-R | GCTGGGTCAGACTGTGAGCAAT |  |  |
|  | *Bbox1*-F | CGGCTCCTCTGCCCTTCA | 113 | 109.46 |
|  | *Bbox1*-R | CGGATACAAGCTACAACTCCCA |  |  |
|  | *BLVRB*-F | TGACGGGGTTAGCGACCTTAC | 128 | 103.32 |
|  | *BLVRB*-R | GTTCAAAACATCTCCCACCACC |  |  |
